# A modified method by differential adhesion for enrichment of bladder cancer stem cells

**DOI:** 10.1590/S1677-5538.IBJU.2015.0409

**Published:** 2016

**Authors:** Yong-tong Zhu, Shi-yu Pang, Yang Luo, Wei Chen, Ji-ming Bao, Wan-long Tan

**Affiliations:** 1 Department of Obstetrics and Gynecology, Center for Reproductive Medicine, Nanfang Hospital, Southern Medical University, Guangzhou, China; 2 Department of Urology, Nanfang Hospital, Southern Medical University, Guangzhou, China

**Keywords:** Urinary Bladder Neoplasms, Neoplastic Stem Cells, Trypsin

## Abstract

**Purpose:**

In a previous study the vaccine was effective against bladder cancer in a mouse model. However, a small portion of tumors regrew because the vaccine could not eliminate bladder cancer stem cells (CSCs). In this study, we showed a modified method for the isolation of bladder CSCs using a combination of differential adhesion method and serum-free culture medium (SFM) method.

**Materials and Methods:**

Trypsin-resistant cells and trypsin-sensitive cells were isolated from MB49, EJ and 5637 cells by a combination of differential adhesion method and SFM method. The CSCs characterizations of trypsin-resistant cells were verified by the flow cytometry, the western blotting, the quantitative polymerase chain reaction, the resistance to chemotherapy assay, the transwell assay, and the tumor xenograft formation assay.

**Results:**

Trypsin-resistant cells were isolated and identified in CSCs characters, with high expression of CSCs markers, higher resistance to chemotherapy, greater migration in vitro, and stronger tumorigenicity in vivo.

**Conclusion:**

Trypsin-resistant cells displayed specific CSCs properties. Our study showed trypsin-resistant cells were isolated successfully with a modified method using a combination of differential adhesion method and SFM method.

## INTRODUCTION

The human interleukin-2 surface modified MB49 bladder cancer cells vaccine induced specific antitumor immunity and was effective against metastatic bladder cancer in our previous study (1). However, a small portion of the mouse bladder tumors underwent regression and regrew after a period of time because the cancer stem cells (CSCs) were not eliminated. Recurrence of solid tumors may be due to the inability of traditional chemotherapy and radiotherapy to eliminate CSCs (2, 3). The vaccine used in our previous study was not the CSCs vaccine and thus could not induce specific immunity directed against CSCs.

It has been found that repeated cycles of differential adhesion could enrich for breast CSCs by 20-fold, and the relation between stem cell properties and adhesiveness has been noted previously in other cancer cells (4). The serum-free culture medium (SFM) method had been used to isolate CSCs from cancer cells, but it was limited due to the deficiency of purity in the CSCs (5). As we known, the combination of the differential adhesion method and SFM method has not been used to enrich the CSCs, which could gain the purity of cell sorting. The enrichment of bladder cancer stem cells would promote development of our vaccine research. Thus, we provide a modified method here by combining the differential adhesion and SFM methods to enrich bladder CSCs.

## MATERIALS AND METHODS

### Cell lines

The murine bladder cancer cell line, MB49, was a gift from Dr. I. C. Summerhayes (1). The human bladder cancer cell lines, EJ and 5637, were provided and preserved in Pathology Lab, Southern Medical University. These cells were cultured in RPMI1640 that contained 10% fetal bovine serum (FBS, Thermo Scientific HyClone, Logan, Utah) at 37°C in a 5%CO_2_ humidified incubator.

### The differential adhesion method

Cells were cultured to confluency in a 6-well plate, washed with phosphate buffered saline (PBS), and digested with trypsin solution (eBioscience, San Diego, California) at 37°C. After several minutes, cells were divided and collected by washing with PBS. Trypsin was added in the attached cells and the digested and collected process repeated several times with the same times. Cells collected after different times were cultured in a 6-well plate for 3 days. Then the trypsin-sensitive cells and trypsin-resistant cells were digested with trypsin again, divided and collected as former, separately. Such step was repeated for 3 cycles. Finally, the trypsin-resistant cells were cultured with SFM in a 6-well plate. By the 15th day, these cells had grown to spheres and were considered to be CSCs.

The constitutes of SFM were RPMI1640, fibroblast growth factor basic (20ng/mL), epidermal growth factor (20ng/mL), B-27 serum-free supplement (20μL/mL), leukemia inhibitory factor (20ng/mL) and bovine serum albumin (4μg/mL).

### Flow cytometry (FCM)

The MB49, EJ and 5637 cells and their relevant CSCs were harvested respectively. They were dissociated in autoMACS running buffer (Miltenyi Biotec, Bergisch Gladbach, Germany), labeled with FITC antiCD44 (Miltenyi Biotec) and PE anti-prominin-1 (Miltenyi Biotec), incubated at 4°C for 20 minutes, and washed twice with PBS. The FITC rat IgG2bκ isotype control (eBioscience) and the PE rat IgG1κ isotype control (eBioscience) were used as a control. The portion of CD133^+^CD44^+^ cells was calculated using a BD FACSAria cell sorter (Becton-Dickinson, San Jose, California).

### Quantitative polymerase chain reaction (qPCR)

Total RNA was isolated using Arcturus PicoPure RNA isolation kit (Arcturus, Life Technologies, CA, USA). The RNA quality was verified using Bioanalyzer RNA Pico Chip (Agilent Technologies, CA, USA). cDNAs were synthesized by reverse transcription using the Superscript III reverse transcriptase (Invitrogen, CA, USA). cDNAs were amplified using SYBR green PCR master mix (Bio-Rad, California) on a 7500 real time PCR system (AB Applied Biosystems, Singapore). The sequences of the primers used are listed in Table-1. GAPDH was used as a control. Normalization and fold changes were calculated using the ΔΔC_t_ method (6).

**Table 1:** Primers of selected genes.

Gene name	Primers (forward/reverse)	Base pairs of product
CD133	F: 5’-CGGGATCCGAAAAACTGATCTGT-3’	615bp
	R: 5’-CCGCTCGAGTTACCTAGTTACTCTCTCC-3’	
CD44	F: 5’-CCCTGCTACCAGAGACCAAGAC-3’	401bp
	R; 5’-GCAGGTTCCTTGTCTCATCAGC-3’	
GAPDH	F: 5’-CCATGGAGAAGGCTGGGG-3’	198bp
	R: 5’-CAAAGTTGTCATCCATGACC-3’	

### Western blotting (WB)

The protein extracts were separated by electrophoresis and transferred to polyvinylidene difluoride membranes (Millipore, MA, USA). Membranes were blocked and incubated using the primary antibody anti-CD133 (Abcam, MA, USA), anti-CD44 (Abcam) and anti-β-actin antibody (Abcam). Then membranes were incubated with anti-mouse secondary antibodies (Abcam) (7). Finally, protein bands were detected using Fluor Chem FC2 (Alpha Innotech, CA, USA) and their intensity was analyzed using the Image Lab software.

### Chemotherapy-resistance ability

The cells were seeded onto a 96-well plate at a density of 1×10^4^ per well. The chemotherapeutic agents paclitaxel (Sigma, MO, USA) and cisplatin (Sigma) were added at different concentrations. After 4 days, CCK-8 was added and the absorbance value was recorded. Cell viability was calculated as the percentage points of the absorbance values in treated wells relative to untreated control wells (6).

### Migration abilities in vitro

Cells were seeded, in pure RPMI1640 (1×10^4^ cells/0.25mL/well), onto the upper well, and a 6.5-mm pore-size polycarbonate membrane chamber was inserted into the transwell apparatus (Costar, MA, USA). RPMI1640 containing 10% FBS was added into the lower well. Cells were incubated and migrated to the bottom surface after 24 hours, fixed, stained, rinsed and examined by inverted microscopy.

### Tumorigenic abilities in vivo

All animal experiments performed were approved by the Ethics Committee of Southern Medical University under Contract 1116904. The MB49, EJ and 5637 cells and their relevant CSCs were injected subcutaneously into 4-week-old nude mice (Guangzhou, China). The volume of the tumor xenograft was observed every week, removed at week 8 and measured. The volume of tumors was measured by using the formula d^2^×D/2, where d and D were the shortest and the longest diameters respectively (8).

#### Statistical analysis

All analyses were performed by the SPSS19.0 software, setting significance at P<0.05. All of the data was expressed as the mean±standard deviation and analyzed using one-way ANOVA.

## RESULTS

### Trypsin-resistant cells separable by differential adhesion method

**Figure 1:**
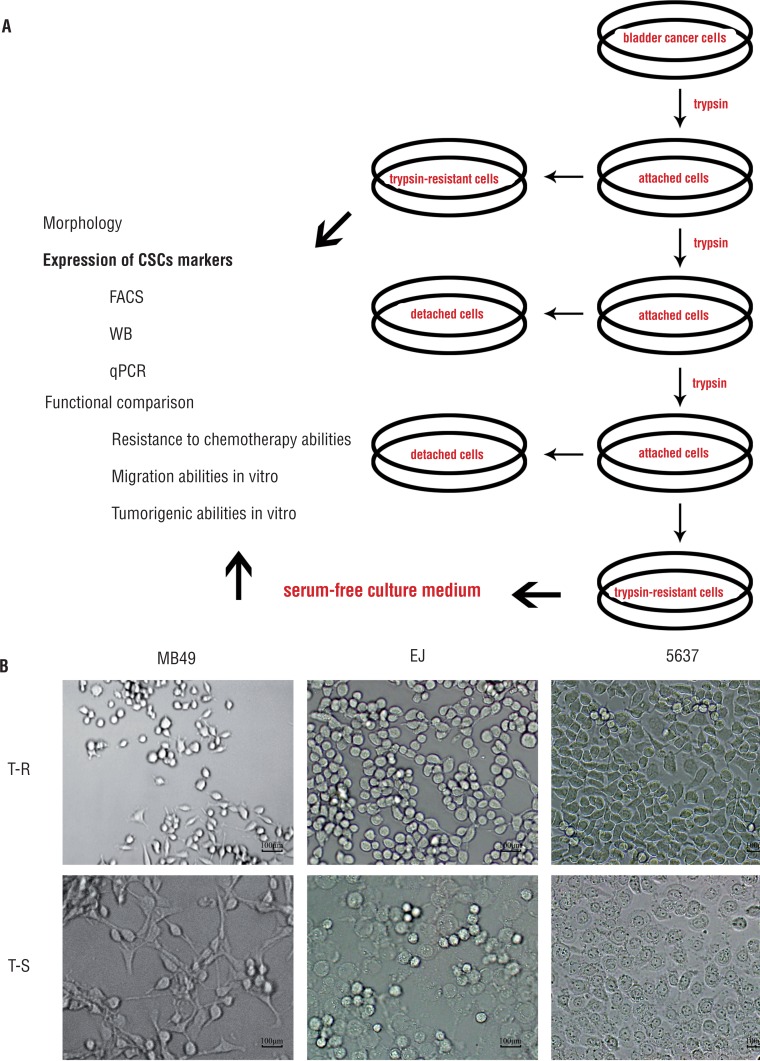
Isolation of cells by differential adhesion method and serum-free culture medium (SFM) method. (A) Diagram illustration to the proposed model for isolation of CSCs by differential adhesion method and sfM method. (B) Morphology of trypsin-resistant cells (T-R) and trypsin-sensitive cells (T-S) in Mb49, EJ and 5637 cells.

The differential adhesion method showed that the digested time of MB49, EJ and 5637 cells was different, average time was 2, 3 and 5 mins, respectively (Figure-1A). It is found that cells detached by fewer time of trypsinization retained original shaped morphologies. On the other hand, cells detached by more time exhibited round shaped morphologies, and cells detached by middle time had mixed morphologies (Figure-1B).

### Characterizations of CSCs

Compared with the trypsin-sensitive cells, trypsin-resistant cells could enrich for CSCs. And these cells cultured with SFM method could gain the purity of CSCs sorting. To confirm this conclusion, it is necessary to identify trypsin-resistant cells in CSCs characters with high expression of CSCs markers, higher resistance to chemotherapy, greater migration in vitro, and stronger tumorigenicity in vivo.

### Expression of CSCs markers

**Figure 2:**
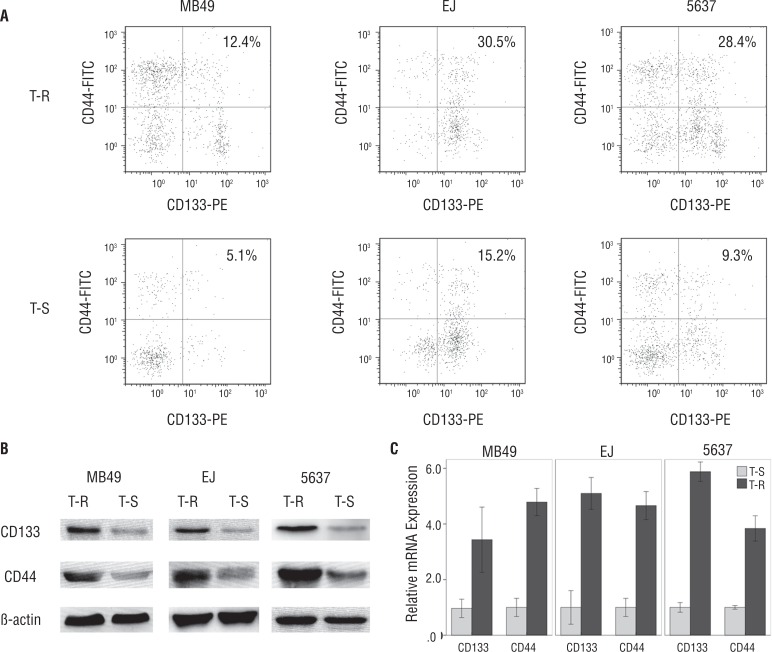
Comparison of specific markers in trypsin -resistant cells (T-R) and trypsin-sensitive cells (T-S) of MB49, EJ and 5637 cells. (A) The FCM analysis revealed that the fraction of CD44^+^CD133^+^ cells in trypsin-resistant cells was more than in trypsin-sensitive cells. (B) The WB analysis indicated that the CD133 and CD44 proteins were abundantly expressed in trypsin-resistant cells, but much less in trypsin-sensitive cells. (C) The qPCR analysis showed that the relative levels of CD133 and CD44 mRNAs in trypsin-resistant cells were higher than those observed in trypsin-sensitive cells.

The FCM analysis revealed that the fraction of CD44^+^CD133^+^ cells in trypsin-resistant cells was more than in trypsin-sensitive cells of MB49, EJ and 5637 cells (Figure-2A). The WB analysis indicated that the CD133 and CD44 proteins were abundantly expressed in trypsin-resistant cells, but much less in trypsin-sensitive cells (Figure-2B). The qPCR analysis showed that the relative levels of CD133 and CD44 mRNAs in trypsin-resistant cells were higher than those observed in trypsin-sensitive cells (Figure-2C).

### Functional comparison

**Figure 3:**
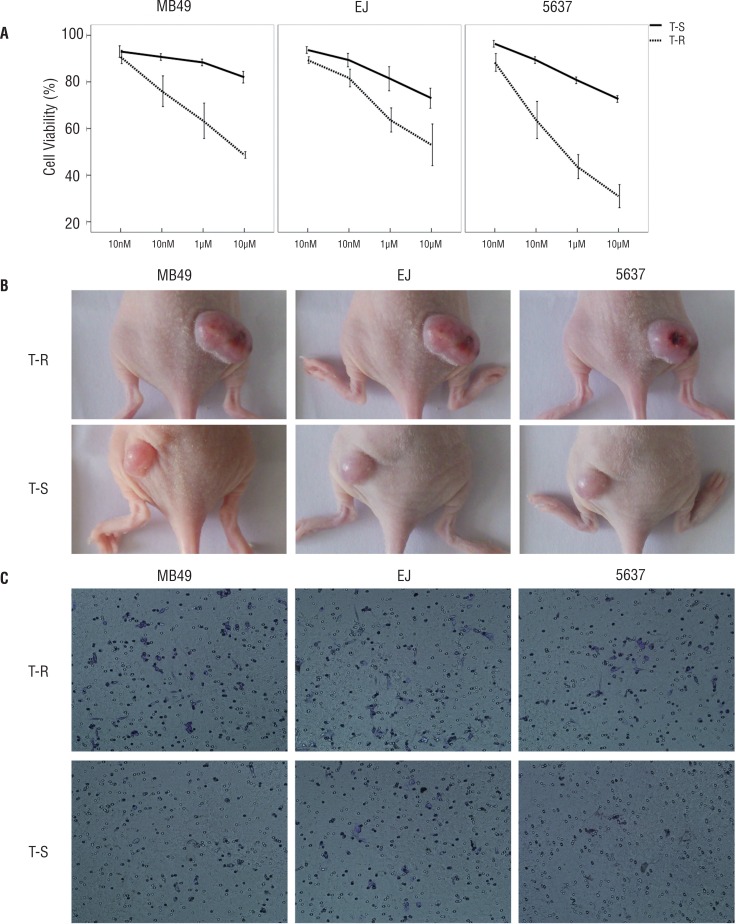
Functional characteristics comparison in trypsin-resistant cells (T-R) and trypsin-sensitive cells (T-S). (A) Comparison of resistance to chemotherapy using CCK-8. Compared to trypsin-sensitive cells, trypsin-resistant cells show higher cell viabilities after treatment with various concentrations of anti-cancer drugs doxorubicin. (B) In the transwell migration assay, the number of invaded trypsin-resistant was higher than that of trypsin-sensitive cells. (C) In xenograft formation experiments, trypsin-resistant produced larger tumor volumes than trypsin-sensitive cells did.

Compared to trypsin-sensitive cells, trypsin-resistant cells displayed higher cell viabilities after being exposed to different concentrations of doxorubicin, which suggested that trypsin-resistant cells had lower susceptibility to traditional anticancer agents (Figure-3A). The results of the transwell migration assay indicated that more trypsin-resistant cells invaded the bottom chamber when compared to trypsin-sensitive cells under the same incubation conditions, which suggested that trypsin-resistant cells had higher invasion ability than trypsin-sensitive cells (Figure-3B). Regarding xenograft formation, trypsin-resistant cells produced tumors with larger volumes than trypsin-sensitive cells did with the same number of injections (Figure-3C).

## DISCUSSION

Enrichment of CSCs was an absolute necessity when using CSCs in vaccine applications. Three methods were mostly used to isolate CSCs from cancers: specific CSCs surface markers, side population cells, and SFM. The shortcoming of these methods was the lack of purity and the purity was not enough for CSCs (5). Repeated cycles of differential trypsinization could gather CSCs by 20-fold in breast cancer cells, keratinocytes and human mammary epithelial cells. So the differential adhesion method was able to isolate CSCs from bladder cancer cells. Considering that the serum caused irreversible differentiation of stem cells, SFM selection might be useful for CSCs expansion and would allow for maintenance of an undifferentiated stem cell status (9). To our knowledge, this is the first report about the isolation and expansion of CSCs via a combination of differential adhesion method and SFM method, which is modified to improve the purity of CSCs.

The CD133 and CD44 markers were used to ascertain CSCs in most tumor tissues (10, 11). Additionally, our study found elevated expression levels of CD133^+^ and CD44^+^ in trypsin-resistant cells. Targeting CD44^+^ and CD133^+^ cancer cells involving a CD133^+^CD44^+^ cell subpopulation might be a way for colorectal cancer therapy (12). Thus CD133^+^CD44^+^ cells may be the concentrated CSC subpopulation in bladder cancer cell populations. Only cells expressing CD133^+^ and CD44^+^ were considered as CSCs by FCM analysis (13). In addition, the expression of both markers was found elevated in trypsin-resistant cells not only at the mRNA expression (qPCR) level, but also at the protein expression (WB) level.

We functionally characterized the trypsin-resistant cells populations by different techniques (14, 15). Specifically, trypsin-resistant cells had a greater ability to penetrate wells. Moreover, although chemotherapy killed most tumor cancer cells, it could not kill CSCs. Additionally, trypsin-resistant cells exhibited a lower sensitivity to doxorubicin, which meet the theory of resistance to chemotherapy (16, 17). Tumorigenicity in nude mice was the standard experiment used to evaluate the tumorigenic ability of CSCs (18). Trypsin-resistant cells had a greater ability to form subcutaneous tumors in nude mice. Taken these above results together, trypsin-resistant cells showed specific CSC properties.

Taken together, this study showed that cultured trypsin-resistant bladder cancer cells displayed specific CSC properties.

## CONCLUSIONS

In conclusion, bladder CSCs were isolated successfully with a modified method using a combination of differential adhesion method and SFM method. Trypsin-resistant cells contained characteristics resembling CSCs such as chemotherapy resistance and in vivo tumorigenic capacity. Trypsin-resistant cells may provide an ideal model for the development of bladder cancer vaccine research.
